# CAMK1D Inhibits Glioma Through the PI3K/AKT/mTOR Signaling Pathway

**DOI:** 10.3389/fonc.2022.845036

**Published:** 2022-04-13

**Authors:** Qianxu Jin, Jiahui Zhao, Zijun Zhao, Shiyang Zhang, Zhimin Sun, Yunpeng Shi, Hongshan Yan, Yizheng Wang, Liping Liu, Zongmao Zhao

**Affiliations:** ^1^ Department of Neurosurgery, The Second Hospital of Hebei Medical University, Shijiazhuang, China; ^2^ Department of Neurology, Beijing Tiantan Hospital, Capital Medical University, Beijing, China; ^3^ Department of Neurosurgery, The Third Hospital of Shijiazhuang City, Shijiazhuang, China

**Keywords:** CAMK1D, glioma, cell proliferation, prognosis, PI3K/AKT/mTOR

## Abstract

Calcium/calmodulin-dependent protein ID (CAMK1D) is widely expressed in many tissues and involved in tumor cell growth. However, its role in gliomas has not yet been elucidated. This study aimed to investigate the roles of CAMK1D in the proliferation, migration, and invasion of glioma. Through online datasets, Western blot, and immunohistochemical analysis, glioma tissue has significantly lower CAMK1D expression levels than normal brain (NB) tissues, and CAMK1D expression was positively correlated with the WHO classification. Kaplan–Meier survival analysis shows that CAMK1D can be used as a potential prognostic indicator to predict the overall survival of glioma patients. In addition, colony formation assay, cell counting Kit-8, and xenograft experiment identified that knockdown of CAMK1D promotes the proliferation of glioma cells. Transwell and wound healing assays identified that knockdown of CAMK1D promoted the invasion and migration of glioma cells. In the above experiments, the results of overexpression of CAMK1D were all contrary to those of knockdown. In terms of mechanism, this study found that CAMK1D regulates the function of glioma cells by the PI3K/AKT/mTOR pathway. In conclusion, these findings suggest that CAMK1D serves as a prognostic predictor and a new target for developing therapeutics to treat glioma.

## Introduction

As the most frequent intracranial malignancy, glioma is characterized with infiltrative growth, high mortality, and easy recurrence (1–3). A clinical trial found that the 5-year survival rate of glioblastoma patients was less than 13% even after receiving the correct treatment including surgery, radiotherapy, and chemotherapy (4, 5). The treatment of glioma patients is becoming more standardized, but the prognosis of highly graded patients is still poor (6, 7). Due to the heterogeneity of glioma gene expression, identifying certain glioma-associated genes as therapeutic targets will help to develop more effective strategies for treating glioma (8, 9).

The development and occurrence of glioma is related to imbalanced expression and mutations of pro-tumor and anti-tumor genes, and therapies targeting molecular markers are a key research direction for the future (10). Previous studies have indicated that CAMK1D plays important roles in the CaMKK-CaMK1 signaling cascade triggered by calcium (11), regulates calcium-mediated granulocyte function and respiratory burst (12), and promotes basal dendrite growth of hippocampal neurons (13). Calmodulin (CaM) regulates the activity of a variety of proteins through binding to calcium, thereby regulating the function of relevant signaling pathways that control a variety of cellular functions (14). In addition, tumor-related studies have found that CAMK1D expression is higher in invasive breast cancer than *in situ* breast cancer and that overexpression of CAMK1D in breast epithelial cells promotes molecular and phenotypic alterations in epithelial–mesenchymal transition (EMT) (15). Overexpression of CAMK1D in lung adenocarcinoma cells promotes cell proliferation but inhibits vascular endothelial cell formation (16, 17). These findings suggest that CAMK1D plays critical roles in the pathogenesis of different cancers. Nevertheless, the clinical significance and function of CAMK1D in glioma remain unknown.

Phosphatidylinositol-3-kinase (PI3K) family is one of the kinases that specifically catalyze the hydroxyl phosphorylation of phosphatidylinositol and produce substances with second messenger effect. The signal pathway composed of PI3K and its downstream molecular signal protein kinase B (AKT)/rapamycin target protein (mTOR) is one of the most important intracellular signal pathways in mammals, which regulates important physiological functions such as cell cycle, protein synthesis, growth, and metabolism. Ribosomal p70S6 kinase (P70S6K) is one of the most characteristic downstream effector molecules of mTOR. The activation of PI3K pathway also increases the phosphorylation of P70S6K (18). It has been demonstrated that calmodulin affects autophagy in prostate cancer cells *via* AKT/mTOR (19) and also participates in PLCγ1-dependent ECM synthesis *via* the mTOR/P70S6K pathway (20). Thus, in this study, we determined if the PI3K/AKT/mTOR pathway plays an important role in CAMK1D regulation of tumor function.

This study focused on the function of CAMK1D in glioma. First, the relative level of CAMK1D in glioma and its relation with overall survival of glioma were analyzed using bioinformatics. We found that downregulation of CAMK1D was associated with poor prognosis in glioma patients. The role of CAMK1D in glioma cell proliferation, invasion, and migration was also explored. Finally, we investigated the potential mechanisms involved in CAMK1D-induced regulation of glioma.

## Materials and Methods

### Bioinformatics Analysis of CAMK1D

Bioinformatics analysis was performed based on 6 glioma cohorts (CGGA, TCGA, Rembrandt, Gravendeel, Phillips and Kamoun) containing clinicopathological and gene expression data obtained from Gliovis (http://gliovis.bioinfo.cnio.es/). We also analyzed the pan-cancer data of CAMK1D in the Cancer Genome Atlas (TCGA), including 33 cancer types using UCSCXenaShiny (https://hiplot.com.cn/advance/ucscxena-shiny). Then, the association between clinical characteristics and CAMK1D expression was explored and visualized through the R package ggplot2. The high- and low-expression groups were distinguished by the median expression of CAMK1D. We subsequently performed survival analysis to compare the overall survival between the low-CAMK1D and high-CAMK1D groups through Survminer R packages.

To determine the exact mechanisms of CAMK1D in glioma, “limma” package in R was applied to detect the differentially expressed genes (DEGs). Then, KEGG (Kyoto Encyclopedia of Genes and Genomes), GSEA (Gene set enrichment analysis), and GO (Gene Ontology) were conducted to investigate potential activating biological function processes or pathways in the low-CAMK1D population.

### Tissue Samples

A total of 80 glioma and normal tissues were obtained from a total of 80 patients treated in The Second Hospital of Hebei Medical University. All samples were confirmed by pathology. Patients who received radiotherapy or chemotherapy before surgery were excluded from this study. The Ethics Committee of The Second Hospital of Hebei Medical University approved this research. The ethics committee waived the requirement of written informed consent for participation.

### Cell Cultures and Reagent

A total of four glioma cell lines (U251, A172, LN229, and U87) and normal human astrocytes (NHAs) were obtained from Procell Life Science. The U251 cells were cultured in RPMI−1640 medium (Gibco™,11875093). The A172 and LN229 cells were cultured in DMEM (Gibco™, 10564011). The U87 cell lines were cultured in MEM (Gibco™, 11090081), while the NHA cells were cultured in astrocyte medium (AM, Sciencell™, #1801). All media were supplemented with 10% FBS (Gibco™, 10099141), 100 U/ml penicillin, and 100 μg/ml streptomycin (Pen-Strep Solution, BI, 2114091). Cells were incubated with 5% CO_2_ at a temperature of 37°C in a standard humidified incubator.

### Cell Transfection

The CAMK1D−coding sequence was synthesized and cloned into the pcDNA 3.1 vector by Hanbio Biotechnology Co., Ltd. to construct a CAMK1D overexpression plasmid (pcCAMK1D). The blank pcDNA3.1 plasmid was used as negative control (NC) plasmid. The siRNA for silencing CAMK1D was designed and synthesized from Thermo Fisher Scientific. The targeting sequence for siCAMK1D was AAGAUGUAGGCAAUCACUCCG. Plasmids (8 μg/10^6^ cells) or small interfering RNA (siRNA) (60 pmol/10^6^ cells) were transfected in glioma cells using Lipofectamine 3000 (L3000015, Invitrogen), according to the product instruction. After 48–72 h, all cells were collected and used in the follow-up experiments.

### RT-PCR

Total RNA was extracted using TRIzol (Invitrogen) and then we used PrimeScript RT reagent (Takara) to reverse transcribe RNA (1 μg) into cDNA according to the instructions. Regarding RT-PCR, the ABI Prism 7500 RT-PCR system (Applied Biosystems) and the SYBR Premix ExTaq (Takara) were used. RT-PCR cycles included pre-denaturation for 30 s at 95°C, denaturation for 5 s at 95°C, annealing for 30 s at 60°C, and extension for 30 s at 72°C, 40 cycles in the last three steps. The primer sequences were as follows (5’−3’): CAMK1D, forward AATGGAGGGCAAAGGAGATGTGATG, and reverse GTA AGGTTTCTGGGCGAGGACTTC; GAPDH, forward GGAGCGAGATCCCTCCAAAAT, and reverse GGCTGTTGTCATACTTCTCATGG. The relative CAMK1D expression was calculated using the 2^−ΔΔCq^ method.

### Immunohistochemistry

Paraffin-embedded tissue specimens (4 μm thick) were sectioned, dewaxed, and rehydrated in gradient ethanol. Then, we add 3% H_2_O_2_ and soak for 10 min to remove endogenous peroxidase. The antigen retrieval was accomplished in 10 mM citrate at 95°C for 20 min. Slides were blocked in goat serum for 60 min and incubated with rabbit anti-CAMK1D antibodies (1:100; ab172618; Abcam) overnight at 4°C. After washes in phosphate buffered saline (PBS), goat anti-rabbit secondary antibody (sp-9001, Zhongshan Golden Bridge) was added for 60 min at room temperature. Subsequently, slides were incubated with horseradish peroxidase (HRP) (sp-9001, Zhongshan Golden Bridge). The slides were lightly counterstained with hematoxylin and dehydrated. Finally, images were captured with Leica DM2000 microscope.

### Western Blot

Tissues and cells of human were lysed in radioimmunoprecipitation assay buffer (Beyotime) containing a protease and phosphatase inhibitor cocktail (K1015, APExBIO). The protein concentration was assayed by the BCA method. The extracted or enriched protein samples were separated by denaturing 10% SDS-polyacrylamide gel electrophoresis and transferred into polyvinylidene fluoride membranes. After being blocked by 5% bovine serum albumin (BSA) for 120 min at room temperature, the membranes were incubated with primary antibodies from abcam and Cell Signaling Technology against GAPDH (ab8245, 1:8,000), mTOR (ab2732, 1:1,500), AKT (ab18785, 1:1,000), p-mTOR (ab109268, 1:1,500), p-AKT (ab38449, 1:1,000), P70S6K (#2708, 1:1,000), p-P70S6K (#9205, 1:1,000), and CAMK1D (ab172618, 1:1,500) overnight at 4°C. After washing three times, the blots were further incubated with goat anti-rabbit IRDye 800CW preadsorbed secondary antibody (1:10,000; Abcam; ab216773). The images were detected with an Odyssey infrared imaging scanner (LI-COR, USA).

### CCK-8 Assay

Cell samples were initially seeded at 100 μl of medium containing 5×10^3^ cells/well on 96−well plates. Then, we measured the cell proliferation rate at 0, 1, 2, 3, and 4 days after transfection. Each well in the 96−well plate was supplemented with 10 μl of CCK-8 (Report) and incubated at 37°C for an additional 2 h. Eventually, the absorbance was measured at 450 nm of 96−well plates and was determined by the use of a strip reader (SpectraMax Plus 384).

### Transwell Migration and Invasion Assays

Seeding of transfected cancer cells in the serum-free medium was performed in the upper chamber of Transwell (8−μm; BD Biosciences) precoated with 40 μl of Matrigel (to assess invasion) or non-coated (to assess migration). Then, 10% FBS containing culture medium was plated into the lower chamber. After incubating for 1 day, cells were removed on the upper chamber with cotton swabs, fixed with 4% paraformaldehyde, and stained in 0.1% crystal violet.

### Colony Formation Assay

Cell samples of glioma were planted at 800 cells/well to the 6-well plates. Following 2 weeks of cell culture, samples were treated with 4% paraformaldehyde and Giemsa stain in succession, and then the total number of colonies was counted with ImageJ (version1.52p).

### Wound Healing

Cell samples of glioma were plated into the six-well plate for treatment and grew up to 90% confluency. Then, we created the scratch with a pipette tip on these cell monolayers. The scratch was photographed with the microscope (Olympus, Japan) at 0 h and 24 h at the same position. Finally, the width of the scratch was analyzed with ImageJ.

### Xenograft Experiment

Glioma cells (5×10^6^) were injected into the subcutis of athymic BALB/c nude mice (4 weeks, male). The length (L) and the width (W) of xenograft were estimated using a vernier caliper at 7, 14, 21, and 28 days. The xenograft volume was calculated by the equation: V = (W^2^ × L)/2. After 28 days, the mice were sacrificed and subcutaneous xenografts were collected, photographed, and weighed. The regulations of the Ethics Committee of the Second Hospital of Hebei Medical University were followed in animal experiments.

### Statistical Analyses

Each experiment was repeated thrice and exhibited as the mean values ± standard deviation (SD). All data were analyzed statistically by Prism 8 (GraphPad Inc, USA) and R software (version 3.6.3). Student’s *t*-test and one-way ANOVA were used in statistical analysis. The prognostic value of patients was shown using the Kaplan–Meier curve. Factors for overall survival were performed using Log-rank test and *p*-values less than 0.05 were judged as statistically significant difference between groups.

## Results

### Pan−Cancer Analysis of CAMK1D Expression

**Figure 1:**
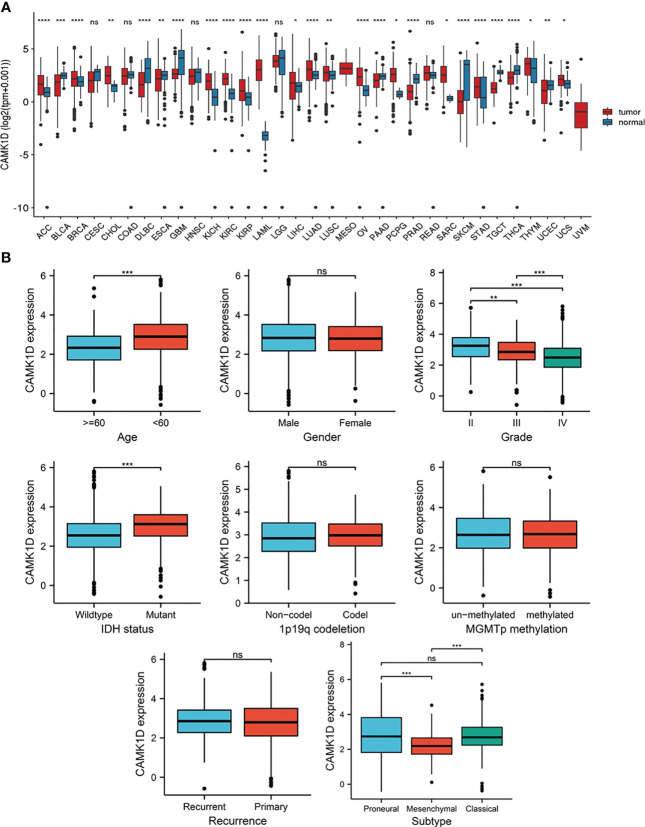
Pan-cancer analysis of CAMK1D expression and the relationship between the expression of CAMK1D and clinicopathological features. **(A)** CAMK1D mRNA expression in pan-cancer dataset of TCGA. **(B)** Expression of CAMK1D in different age, genders, WHO grades, IDH status, 1p19q codeletion, recurrence, MGMTp methylation, and subtypes of glioma. Please note the CAMK1D gene expression level changes in different tumor tissues. Furthermore, CAMK1D expression level was low in glioma tissue with higher WHO grade patients. ns, no significance; *p < 0.05 < **p < 0.01 < ***p < 0.001 < ****p < 0.0001.

Pan-cancer analysis showed that CAMK1D expression levels were significantly different between multiple tumor tissue and adjacent tissues (or GTEx) (**Figure 1A**). Expression of CAMK1D was lower in BLCA, DLBC, GBM, LGG, PAAD, PRAD, SKCM, TGCT, THCA, and UCEC than in adjacent tissues, whereas CAMK1D expression levels were significantly higher in tumor of ACC, BRCA, CHOL, ESCA, KICH, KIRC, KIPR, LAML, LIHC, LUSC, LUAD, OV, PRAD, SARC, PCPG, STAD, UCS, and THYM than in adjacent tissues.

### The Expression of CAMK1D Is Related to Clinical Characteristics of Glioma Patients

To determine the role of CAMK1D in tumor pathogenesis and development, we studied the relationship between CAMK1D protein and clinicopathological features, including gender, age, recurrence, WHO grade, 1p19q codeletion, subtype, IDH status, and MGMTp methylation in the CGGA dataset. Our results show that the expression of CAMK1D in glioma tissues was decreased in aged and higher WHO grade patients. In addition, CAMK1D expression levels were different under different subtypes and different IDH mutations in different states (**Figure 1B**).

**Figure 2:**
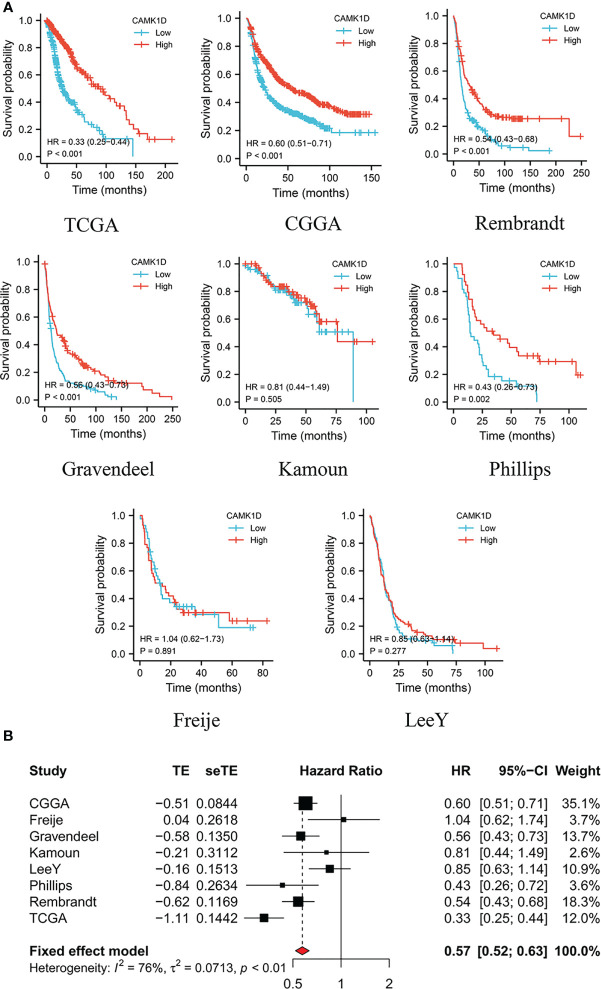
Decreased expression of CAMK1D predicts short survival time of glioma patients. **(A)** Survival curves of CAMK1D in various datasets of glioma showing that glioma patients with lower expression level of CAMK1D have shorter survival time than patients with higher expression levels. **(B)** The forest plot was presented in the RRs for glioma patients with high expression of CAMK1D compared to low expression.

The patients were divided into low- and high-CAMK1D groups based on the median expression of CAMK1D. The survival analysis indicated that glioma patients with lower expression of CAMK1D in TCGA (HR: 0.33, 95% CI 0.25–0.44), CGGA (HR: 0.60, 95% CI 0.51–0.71), Rembrandt (HR: 0.54, 95% CI 0.43–0.68), Gravendeel (HR: 0.56, 95% CI 0.43–0.73), Kamoun (HR: 0.81, 95% CI 0.44–1.49), Phillips (HR: 0.43, 95% CI 0.26–0.73), Freije (HR: 1.04, 95% CI 0.62–1.73), and LeeY (HR: 0.85, 95% CI 0.63–1.14) exhibited significantly shorter survival time than patients with higher expression, while patients from the Kamoun cohort displayed the same trend with no statistical significance (**Figure 2A**). To show the reliability of our results, a meta-analysis was performed to gather the HR of the eight glioma datasets, and results also confirmed that low-CAMK1D patients displayed shorter OS time compared to high-CAMK1D patients (RR:0.5, 95% CI 0.52–0.63, **Figure 2B**).

### CAMK1D Low Expression in Glioma Predicted Poor Survival of Glioma Patients

**Figure 3:**
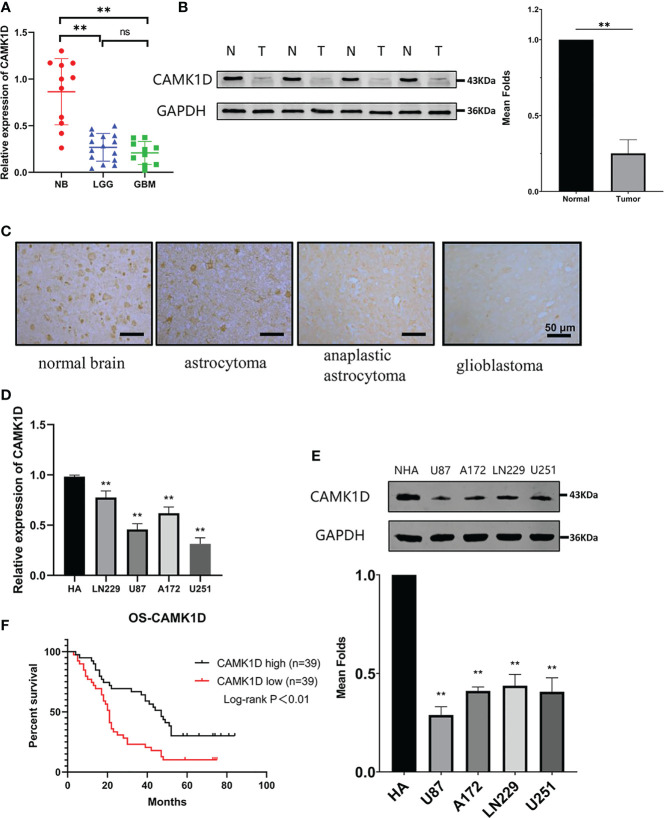
CAMK1D expression in tissues and cells. **(A)** CAMK1D mRNA expression was signficantly decreased in glioma samples compared with normal brain samples (NB = 11; LGG = 15; GBM = 10). **(B)** CAMK1D protein expression was significantly decreased in four glioma samples compared with four normal brain samples with Western blot analysis. **(C)** Immunohistochemistry showed that the expression of CAMK1D immunoreactivities were different in normal brain tissue, astrocytoma, anaplastic astrocytoma, and glioblastoma. **(D, E)** CAMK1D expression levels in HA cell lines and four glioma cell lines were determiend by RT-PCR and Western blot. Both mRNA and protein levels were signficantly decreased in the 4 glioma cell lines compared with HA cell lines. **(F)** Kaplan–Meier curves for overall survival of glioma patients based on expression of CAMK1D protein (78 glioma samples) showing that low-level CAMK1D expression was closely associated with the poor overall survival of glioma patients. ns, no significance; **p < 0.01.

To further explore the functions of CAMK1D in glioma, CAMK1D expression was estimated in glioma tissues compared with normal brain tissues using Western blot, IHC, and RT−qPCR. The results indicated that the expression of CAMK1D was strikingly downregulated in glioma tissues from patients at all 4 WHO grades compared with normal brain tissues (**Figures 3A-C**). The expression levels were decreased progressively as the grades increased. Similarly, the expressions of CAMK1D in 4 glioma cell lines were significantly lower than the NHA (**Figures 3D, E**). These results suggest that CAMK1D may be a cancer suppressor gene in glioma. The data of Kaplan–Meier survival were obtained from the medical records of glioma patients in The Second Hospital of Hebei Medical University hospital. According to the median cutoff of the expression of CAMK1D, the patients were separated into two groups. The result revealed that low CAMK1D expression was significantly related with the poor overall survival of glioma patients (**Figure 3F**).

### CAMK1D Inhibits Glioma Cell Proliferation *In Vitro*

**Figure 4:**
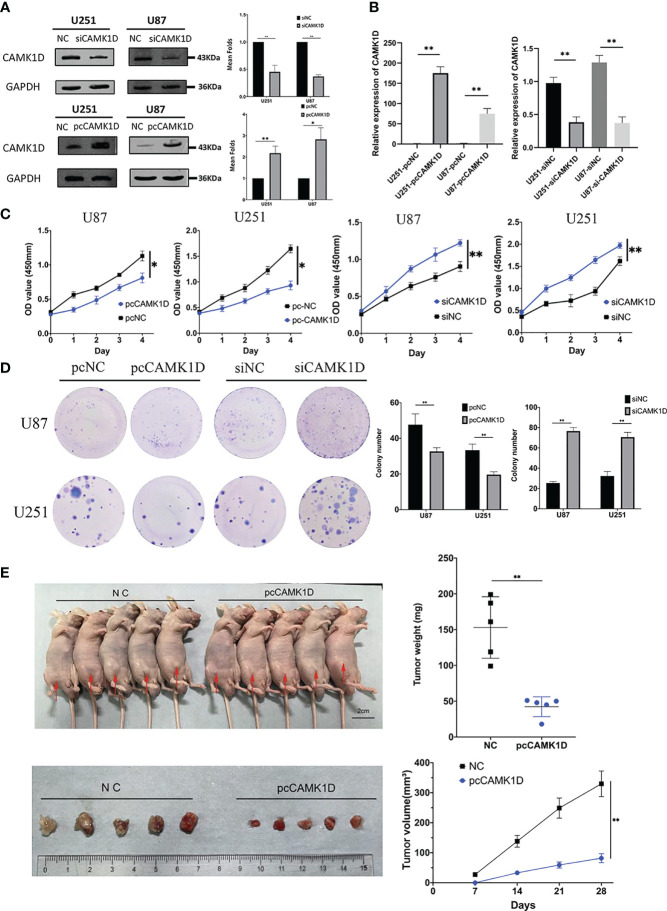
CAMK1D inhibits glioma cell proliferation. **(A, B)** The protein and mRNA levels of CAMK1D were able to be downregulated and overexpressed in glioma cells by siCAMK1D and paCAMK1D. **(C)** CCK8 assays measured the effect of CAMK1D on the growth of glioma cells. **(D)** Colony formation assay used to verify the influence of CAMK1D on glioma cell proliferation. **(E)** The tumor growth rate *in vivo* was inhibited after injection of U251 cells overexpressing CAMK1D. *p < 0.05; **p < 0.01.

We detected the expression levels of CAMK1D in U251 and U87 cells that were transfected with plasmid or siRNA for 2 days using RT−qPCR and Western blot. The mRNA and protein levels of CAMK1D were notably upregulated in glioma cells transfected with CAMK1D plasmids and were downregulated in glioma cells transfected with siCAMK1D, compared to the pcNC (transfected an empty vector) and siNC (transfected a scramble siRNA) (**Figures 4A, B**). As evidenced by CCK-8 assay and colony formation assay, CAMK1D overexpression significantly inhibited the proliferation of cells; on the contrary, CAMK1D knockdown resulted in the opposite (**Figures 4C, D**). Proliferation of cells were strengthened by CAMK1D knockdown compared with cells in the siNC group.

### Overexpression of CAMK1D Inhibits the Growth of Glioma Cells *In Vivo*

Based on the experimental results *in vitro*, we inoculated pcCAMK1D and pcNC-treated U251 cells, subcutaneously into immunodeficient mice to establish a tumor transplantation model. The growth of tumors in two groups showed that tumors in the CAMK1D overexpression group grew significantly slower compared with the control group. Twenty-eight days after U251 cell injection, tumors were excised and weighed. The mean volume was 44.69 ± 18.64 mm^3^ in the CAMK1D overexpression group and 172.53 ± 73.32 mm^3^ in the control group. The mean mass weight was 42.40 ± 13.83 mg in the CAMK1D overexpression group and 153.00 ± 43.03 mg in the control group. There were significant differences in tumor weight and volume between the transfected and control groups (**Figure 4E**). These *in vivo* findings showed that CAMK1D inhibited the proliferative ability of U251 glioma cells, which was consistent with our findings in *in vitro* experiments.

### CAMK1D Inhibits Glioma Cell Invasion and Metastasis *In Vitro*

**Figure 5:**
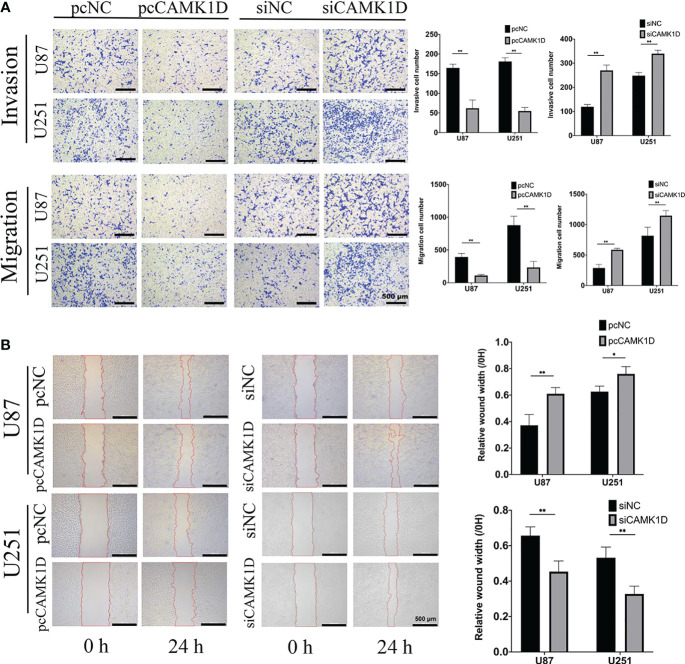
CAMK1D inhibits the invasion and migration of glioma cells. **(A)** Transwell assays revealed that CAMK1D overexpression reduced the invasion and migration of U87 and U251 glioma cells. The invasive cell numbers and migration cell numbers of U87 and U251 cell lines were significantly decreased by overexpression of CAMK1D with pcCAMK1D. **(B)** Wound healing assay showed that overexpression of CAMK1D reduced the relative wound width in U87 and U251 glioma cells. *p < 0.05; **p < 0.01.

To detect the role of CAMK1D in cell invasion and migration, we used scratch wound healing assays, transwell invasion, and migration assays. The results revealed that glioma cell invasion and migration were weakened by CAMK1D overexpression. Meanwhile, cell migration and invasion were strengthened by CAMK1D knockdown compared with cells in the siNC group (**Figures 5A, B**). The *in vitro* data suggest that CAMK1D represses glioma cell proliferation, invasion, and migration abilities.

### Conformation of Differentially Expressed Genes

**Figure 6:**
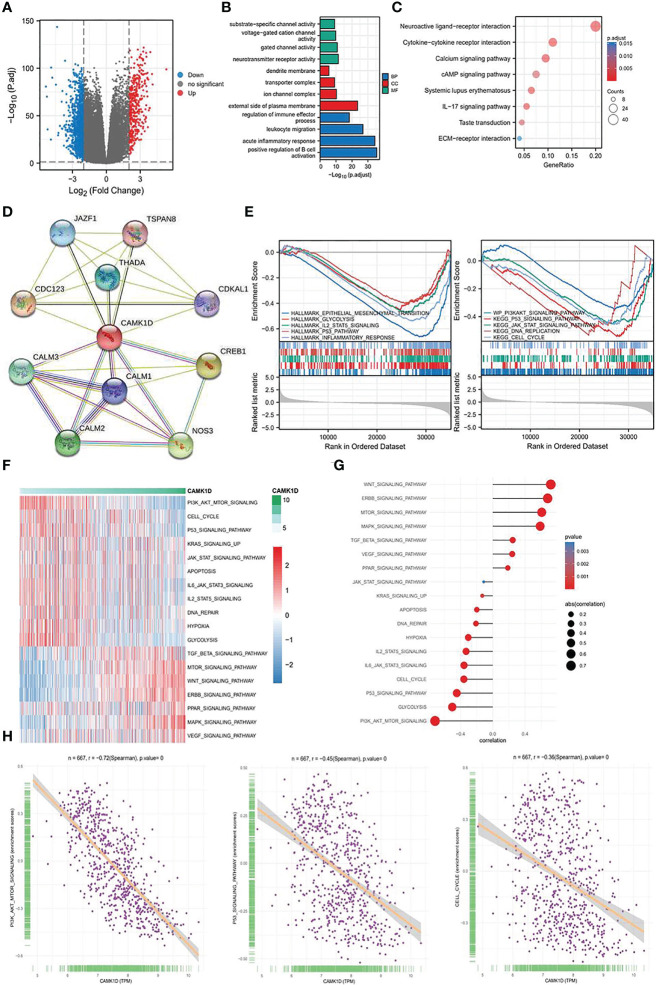
Determination of the biological mechanism and processes affected by CAMK1D. **(A)** The Volcano plot showed the DEGs in low- and high-CAMK1D groups. **(B)** Analysis of GO enrichment for the DEGs. **(C)** Analysis of KEGG pathway enrichment for the DEGs. **(D)** The PPI network of CAMK1D constructed by the STRING database. **(E)** GSEA between the low- and high-risk group **(F)** The heatmap showed the GSVA scores of 18 pathways in low- and high-risk populations. **(G)** The association graph between GSVA and risk scores of 18 pathways. **(H)** The correlation between the PI3K/AKT/mTOR signaling, P53 signaling, cell cycle, and the expression of CAMK1D.

To understand the potent mechanisms underlying the role of CAMK1D in regulating glioma proliferation, invasion, and migration, we analyzed DEGs between high and low CAMK1D groups in TCGA. Log_2_ fold-change (FC) > 2 and *p*-value less than 0.05 were recognized as screening crit eria, which contained 1,043 downregulated genes and 380 upregulated genes (**Figure 6A**). Enrichment analysis was performed, and the DEGs were mainly enriched in substrate-specific channel activity, dendrite membrane, regulation of immune effector process, neuroactive ligand–receptor interaction, and the PI3K/Akt/mTOR signal pathway (**Figures 6B, C**). Another 10 genes were identified by the STRING database as CAMK1D-related genes with significant interactions, including JAZF1, TSAN8, CDC123, THADA, CDKAL1, CALM3, CALM1, CREB1, CALM2, and NOS3. The PPI network of CAMK1D and CAMK1D-related genes was constructed and visualized by the STRING database (**Figure 6D**). Then, we used GSVA and GSEA methods to predict the potential carcinogenic pathway of CAMK1D. We found that many different signaling pathways were significantly related to CAMK1D expression (**Figures 6E–G**), such as EMT, glycolysis, p53 signaling pathways, and WNT signaling pathways. Notably, both analyses showed that the PI3K/AKT/mTOR signaling pathway were significantly enriched in tumor-related pathways, and the activation of this pathway was negatively correlated with CAMK1D expression (**Figure 6H**).

### CAMK1D Inhibits Proliferation, Invasion, and Migration *In Vitro* Through the PI3K/Akt/mTOR Pathway

**Figure 7:**
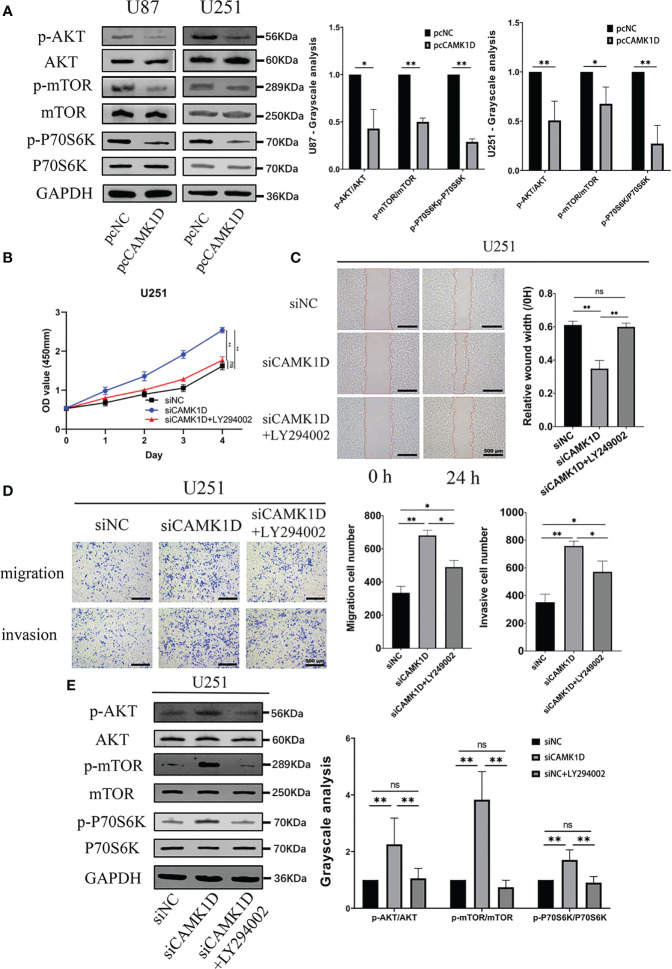
CAMK1D level changes on the PI3K/AKT/mTOR pathway. **(A)** Compared with the control, the expression of p-AKT and p-mTOR was significantly decreased after CAMK1D overexpression using pcCAMK1D plasmid construction in U87 and U251 cells. **(B–D)** LY294002 reversed the increases in cell proliferation, migration, and invasion induced by the decrease in CAMK1D using siCAMK1D in U87 and U251 cells. **(E)** LY294002 reversed the decrease of p-AKT and p-mTOR expression by siCAMK1D. ns, no significance; *p < 0.05; **p < 0.01.

Then, we determined the effect of CAMK1D overexpression on PI3K/AKT/mTOR pathway in glioma cells. Western blot analysis showed that overexpression of CAMK1D downregulated p-Akt, p-mTOR, and p-P70S6K, and decreased p-Akt/Akt, p-mTOR/mTOR, and p-P70S6K/P70S6K ratios in glioma cells (**Figure 7A**). Moreover, the involvement of this pathway in glioma cells was analyzed by applying PI3K/Akt/mTOR pathway inhibitor LY294002. By using CCK-8, transwell, and wound healing assay, we determined whether inhibition of the PI3K/AKT/mTOR signaling pathway by LY294002 reversed the inhibition of cell proliferation, invasion, and migration induced by CAMK1D overexpression. These results obtained from CCK-8, transwell, and wound healing assay all indicated that LY294002 reversed the promotion of cell proliferation, invasion, and migration by siCAMK1D (**Figures 7B–E**). These findings support the notion that CAMK1D regulates glioma cell proliferation, migration, and invasion through the PI3K/AKT/mTOR pathway.

## Discussion

CAMK1D is widely present in a variety of cell types and plays an important role in control of formation of synapses, growth cone movement and axon growth, aldosterone synthase expression, visual signaling processes, and the cell cycle (21–26). Based on bioinformatics analysis and screening, our study was the first to determine the role of CAMK1D in cell proliferation, migration, and invasion of glioma cells. We found that CAMK1D expression was significantly downregulated in glioma cell lines and glioma tissues from patients. However, based on the tumor expression data in the TCGA and GTEx databases, the expression level of CAMK1D in various tumors is not consistent with its corresponding normal tissues. The above findings suggest that CAMK1D probably plays different functions in different cancer types. In addition, CAMK1D expression in the database was associated with overall survival in patients with glioma. This implies that whether CAMK1D can be used as a prognostic marker for glioma patients needs to be studied in a larger collection of glioma specimens.

We found that overexpression of CAMK1D can inhibit the proliferation, migration, and invasion of glioma cells, whereas knockdown of camk1d using siRNA produced opposite effects. This is not identical to the previously reported function of CAMK1D in other cancers. For example, CAMK1D promotes the proliferation of breast cancer through CERB/CCND1 (15), inhibits the angiogenesis of lung adenocarcinoma by HH3 (17), enhances the resistance of multiple myeloma to T cells by phosphorylation of caspase-3 and caspase-6 (27), and inhibits the apoptosis of human choroidal trophoblastic cells (28). This reinforces the fact that camk1d plays different functions in different tumor types. The reason may be that the targeting mechanism of multifunctional kinases allows the same kinases in different cells to respond differently to stimulation and produce different functions (29–31). The function of CAMK1D-mediated depends on the different activation states and binding substrates in different cancers, thus affecting the function and signaling pathways involved in the substrates. Moreover, the different microenvironment of kinase will also affect its conformation and function. Thus, other functions of CAMK1D in glioma needs to be further studied.

To investigate the signaling pathway involved in the regulatory mechanism of CAMK1D in glioma, we predicted and determined that the PI3K/AKT/mTOR pathway was critically involved in the inhibitory effect of CAMK1D in glioma. It has been shown that abnormal activation of the PI3K/AKT/mTOR pathway is associated with many illnesses, including tumorigenesis (32, 33). In this study, we examined the protein levels of Akt/p-AKT, mTOR/p-mTOR, and P70S6K/p-P70S6k. We found that overexpression of camk1d significantly inhibited p−AKT, p-mTOR, and p-P70S6k. Block the PI3K/AKT/mTOR pathway in U251 cells using LY294002 reversed CAMK1D knockdown-mediated changes in cell function. These data suggest that CAMK1D inhibits cell proliferation, invasion, and migration by regulating the PI3K/AKT/mTOR signaling pathways.

Our findings were the first research report showing that CAMK1D overexpression inactivated the PI3K/AKT/mTOR signaling pathway. However, the limitation of this study is that we only studied the PI3K/Akt/mTOR signaling pathway. The regulation of CAMK1D in glioblastoma is a complex network involving multiple genes. In this study, we did not determine whether CERB or HH3 pathway was involved in CAMK1D overexpression-induced inhibition of glioma (15). In addition, we did not examine whether CAMK1D has other functions in glioma, such as reducing apoptosis, affecting EMT, and autophagy. Thus, we cannot rule out the possibility that other pathways are involved in the effect of CAMK1D in glioma, which deserves further investigation in our future studies. Since Ca^2+^ directly regulates CAMK1D activity (34, 35), Ca^2+^ dynamics, especially the frequency, amplitude, and duration of intracellular Ca^2+^ concentration changes, is a potential mechanism to regulate CAMK1D activity.

## Conclusion

In summary, we found that CAMK1D promotes glioma cell proliferation, migration, and invasive processes through activation of the PI3K/AKT/mTOR signaling pathway. These findings suggest that CAMK1D expression is downregulated in glioma and can be used as a prognostic indicator for glioma patients.

## Data Availability Statement

The original contributions presented in the study are included in the article/supplementary material. Further inquiries can be directed to the corresponding authors.

## Ethics Statement

The studies involving human participants were reviewed and approved by the Ethics Committee of the Second Hospital of Hebei Medical University. The ethics committee waived the requirement of written informed consent for participation. The animal study was reviewed and approved by the Ethics Committee of the Second Hospital of Hebei Medical University.

## Author Contributions

QJ and JZ conducted experiments, analyzed and interpreted data, and wrote the original draft. ZJ Zhao performed the bioinformatics analysis. SZ, YS, and HY were involved in animal experiments. YW collected the samples and clinical data. ZS created tables, graphs, and figures. ZM Zhao and LL conceived the idea for the project, revised the paper, and received funding for the project. All authors contributed to the article and approved the submitted version.

## Funding

This research was supported by the National Natural Science Foundation of China (81870984); the National Key R & D Program Intergovernmental Cooperation on International Scientific and Technological Innovation of the Ministry of Science and Technology of China (2017YFE0110400); the Hebei Natural Science Foundation General Project—Beijing-Tianjin-Hebei Basic Research Cooperation Project (H2018206675); the Special Project for the Construction of Hebei Province International Science and Technology Cooperation Base (193977143D); the Government-funded Project on Training of outstanding Clinical Medical Personnel and Basic Research Projects of Hebei Province in the Year of 2017; and the Government-funded Project on Training of Outstanding Clinical Medical Personnel and Basic Research Projects of Hebei Province in the Year of 2019.

## Conflict of Interest

The authors declare that the research was conducted in the absence of any commercial or financial relationships that could be construed as a potential conflict of interest.

## Publisher’s Note

All claims expressed in this article are solely those of the authors and do not necessarily represent those of their affiliated organizations, or those of the publisher, the editors and the reviewers. Any product that may be evaluated in this article, or claim that may be made by its manufacturer, is not guaranteed or endorsed by the publisher.
